# MCAM: Multiple Clustering Analysis Methodology for Deriving Hypotheses and Insights from High-Throughput Proteomic Datasets

**DOI:** 10.1371/journal.pcbi.1002119

**Published:** 2011-07-21

**Authors:** Kristen M. Naegle, Roy E. Welsch, Michael B. Yaffe, Forest M. White, Douglas A. Lauffenburger

**Affiliations:** 1 Department of Biological Engineering, Massachusetts Institute of Technology, Cambridge, Massachusetts, United States of America; 2 Koch Institute for Integrative Cancer Research, Massachusetts Institute of Technology, Cambridge, Massachusetts, United States of America; 3 Sloan School of Management, Massachusetts Institute of Technology, Cambridge, Massachusetts, United States of America; 4 Department of Biology, Massachusetts Institute of Technology, Cambridge, Massachusetts, United States of America; University of Virginia, United States of America

## Abstract

Advances in proteomic technologies continue to substantially accelerate capability for generating experimental data on protein levels, states, and activities in biological samples. For example, studies on receptor tyrosine kinase signaling networks can now capture the phosphorylation state of hundreds to thousands of proteins across multiple conditions. However, little is known about the function of many of these protein modifications, or the enzymes responsible for modifying them. To address this challenge, we have developed an approach that enhances the power of clustering techniques to infer functional and regulatory meaning of protein states in cell signaling networks. We have created a new computational framework for applying clustering to biological data in order to overcome the typical dependence on specific *a priori* assumptions and expert knowledge concerning the technical aspects of clustering. Multiple clustering analysis methodology (‘MCAM’) employs an array of diverse data transformations, distance metrics, set sizes, and clustering algorithms, in a combinatorial fashion, to create a suite of clustering sets. These sets are then evaluated based on their ability to produce biological insights through statistical enrichment of metadata relating to knowledge concerning protein functions, kinase substrates, and sequence motifs. We applied MCAM to a set of dynamic phosphorylation measurements of the ERRB network to explore the relationships between algorithmic parameters and the biological meaning that could be inferred and report on interesting biological predictions. Further, we applied MCAM to multiple phosphoproteomic datasets for the ERBB network, which allowed us to compare independent and incomplete overlapping measurements of phosphorylation sites in the network. We report specific and global differences of the ERBB network stimulated with different ligands and with changes in HER2 expression. Overall, we offer MCAM as a broadly-applicable approach for analysis of proteomic data which may help increase the current understanding of molecular networks in a variety of biological problems.

## Author Summary

Proteomic measurements, especially modification measurements, are greatly expanding the current knowledge of the state of proteins under various conditions. Harnessing these measurements to understand how these modifications are enzymatically regulated and their subsequent function in cellular signaling and physiology is a challenging new problem. Clustering has been very useful in reducing the dimensionality of many types of high-throughput biological data, as well inferring function of poorly understood molecular species. However, its implementation requires a great deal of technical expertise since there are a large number of parameters one must decide on in clustering, including data transforms, distance metrics, and algorithms. Previous knowledge of useful parameters does not exist for measurements of a new type. In this work we address two issues. First, we develop a framework that incorporates any number of possible parameters of clustering to produce a suite of clustering solutions. These solutions are then judged on their ability to infer biological information through statistical enrichment of existing biological annotations. Second, we apply this framework to dynamic phosphorylation measurements of the ERBB network, constructing the first extensive analysis of clustering of phosphoproteomic data and generating insight into novel components and novel functions of known components of the ERBB network.

## Introduction

Large and complex high-throughput proteomic experimental studies are becoming more accessible through the use of powerful, swiftly developing platforms such as mass spectrometry (MS), flow cytometry (FC), and various kinds of protein microarrays (PMA) [1]–[3]. As one particular example of increasing attention, there has been an explosion in large-scale datasets for receptor tyrosine kinase (RTK) network signaling by the combination of protein post-translational modification enrichment followed by quantitative MS methods [4]. In receptor tyrosine kinase (RTK) networks, such as those activated by the ERBB family of receptors, phosphorylation plays a central role in the translation of extracellular cues into phenotypic changes, such as differentiation, proliferation, and migration [5]. Phosphorylation on proteins in the RTK network induce a variety of signaling events including protein-protein interactions, enzymatic activation and inactivation, and cellular localization changes, such as translocation to the nucleus or recruitment to the plasma membrane [6]–[8]. Understanding RTK networks, and the phosphorylation that occurs within them, will be essential for understanding RTK signaling in normal and dysregulated conditions. Mass spectrometry measurement of phosphorylation events in cellular signaling networks is greatly increasing our understanding of the specific modifications occurring in the cell as well as their relative changes in response to network perturbations, such as ligand stimulation or kinase inhibition. However, the pace of identification of phosphorylation sites in cellular networks has outstripped our ability to understand the function and regulation of the measured phosphorylation sites as evidenced by the sharp increase in phosphorylation database repository sizes, such as Phospho.ELM [9]. Unsupervised computational learning methods, applied to quantitative phosphoproteomic data, provides one method by which to infer function and regulation of phosphorylation within cell signaling networks. Previous studies have shown success in application of unsupervised learning to phosphoproteomic data [10]–[12], however each has presented the results of a single clustering solution, and to date, no extensive study of the relationship between unsupervised learning and phosphoproteomic data has been produced. Phosphoproteomic data represents a new challenge in unsupervised learning and currently, no gold standard exists as a method for judging the success of a clustering solution of phosphoproteomic data.

Unsupervised learning approaches, such as various types of clustering algorithms, have been heavily utilized to productive effect in the biological community in application to other kinds of high-throughput biomolecular measurements including gene sequence [13], gene expression [14], and metabolomics [15]. Unsupervised learning algorithms, often referred to as clustering, seek to group components of a multidimensional dataset into clusters where intra-cluster differences are minimized and inter-cluster differences are maximized. Successful application of clustering has demonstrated its usefulness in reducing dataset dimensionality and providing biological hypotheses through the use of inference. Application of unsupervised learning to biological datasets is extensive and includes a seemingly endless option of algorithms, such as Kmeans [14], hierarchical clustering [16], self organizing maps [17], and affinity propagation [18]. Since most clustering algorithms base clustering solutions on the similarity, or dissimilarity, between two vectors of measurements based on a distance metric, the metric used will have an impact on the final solution as will any alteration applied to the vectors by data transformations. Several studies have examined the effect different distance metrics [19]–[21] and data transformations [21], [22] have on clustering solutions of experimentally-derived data. In addition to choosing an appropriate algorithm, distance metric, and data transformation, scientists are also faced with having to determine a suitable number of clusters (K) in which to partition their dataset, since few algorithms incorporate concurrent optimization of K. A variety of methods for determining the natural cluster structure of a dataset have been proposed, see [23] for an example of their comparative performances using a set of microarray experiments. Taken as a whole, the historical application of unsupervised learning to microarray data, as well as other large biological datasets, indicates a vast landscape of decisions required to apply unsupervised learning effectively. We view this vastness as offering an opportunity for enhanced capability to gain biochemical and biological insights, via an approach that takes advantage of the diversity by seeking consistencies and contrasts.

In this work we create a framework, Multiple Clustering Analysis Methodology (MCAM), in which one can apply a vast array of algorithms, distance metrics, data transformations, and cluster set sizes, in a combinatorial fashion, i.e. by the exhaustive combination of all chosen parameters, to a biological dataset and then subsequently compile and evaluate the outcome of all solutions. In this framework, enrichment for biological terms within clustering partitions, relative to the dataset, gives us a metric for evaluating the success of any particular clustering implementation. The use of this framework allows scientists to apply unsupervised learning in a way that requires no *a priori* knowledge or assumptions regarding the most useful clustering algorithm, distance metric, data transformation, or set size. We apply MCAM to diverse phosphoproteomic time-course datasets arising from studies of the ERBB network, where interesting biological enrichment includes Gene Ontology terms, protein domains, kinase and phosphopeptide binding domain predictions, and amino acid sequence motifs. We explore results from these analyses for relationships between biological metrics and the parameters of clustering and develop methods for combining results of clustering to produce robust biological hypotheses and inference. Using this information we explore the difference between two independent measurements of ERBB signaling and find interesting modules of signaling that may be responsible for the migratory potential of HER2 amplification in cancer. The implementation of MCAM was done through a mix of Matlab and Perl scripts. The Perl programs for analyzing enrichment have been incorporated directly into the PTMScout interface (http://ptmscout.mit.edu) [24] and the Matlab tools to generate MCAM cluster sets and analyze resulting enrichment are available for download from PTMScout as well. PTMScout is an open-access web and data resource that contains experimental information concerning protein phosphorylation and lysine acetylation, tools for analyzing proteomic datasets, and the ability for users to directly load new experiments for analysis and data-sharing purposes.

## Results

### Overview of methodology

**Figure 1:**
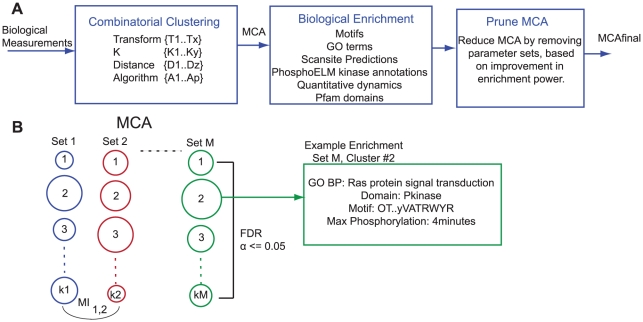
Multiple Clustering Analysis Method. A) MCAM begins with clustering a biological dataset through the combinatorial application of a set of clustering parameters, followed by biological enrichment testing in various categories of information. Following this, the enrichment is used to prune those parameters that contribute little biological information. B) The depiction of an MCA, which contains M sets, with each set having some number of k clusters and produced by a particular combination of clustering parameters. Biological enrichment is corrected for multiple hypothesis testing by using the False Discovery Rate procedure across a set and within a category of biological information. Mutual Information can be used to compare the resulting clustering solution between any two sets.

The MCAM framework is depicted in Figure 1. Our specific interest was to cluster dynamic phosphorylation measurements of the ERBB network in order to find phosphorylation events with similar temporal dynamics leading to possible hypotheses regarding shared regulation or shared functionality. In the MCAM framework, a biological dataset, here dynamic phosphorylation measurements, is first subjected to a set of data transformations, such as log transformation or mean-centering; this transformed data is then clustered via the exhaustive combination of different algorithms, distance metrics, and solution set sizes (K). In our implementations, this produces on the order of 1,500 to 2,500 clustering sets, which we refer to as an MCA. Each set in an MCA represents the complete clustering solution of a single combination of clustering parameters. Next, we calculate enrichment in all biological metric terms, as well as markers for time dynamics, Table 1, within each cluster, compared to the full dataset, using the hypergeometric function and the False Discovery Rate, FDR [25], procedure to correct for multiple hypothesis testing for every metric across a set, Figure 1B. Enrichment in biological metrics will give us a method by which we can judge the fitness of a clustering solution for known biological information, where those categories include information about structure and function of a protein as well as information about the regulation and function of the particular phosphorylation sites, Table 1. Finally, we test, for every parameter of clustering, the impact on the biological information if all sets with that parameter are removed. Parameters are removed from the final set when the removal of that parameter results in a significant improvement of overall biological enrichment and does not significantly decrease the enrichment of any single category of biological information. This pruning, or parameter refinement, allows us to remove clustering solutions which, according to the current biological metrics, are ineffective at producing solutions of biological import. We call this post-pruned set of solutions 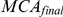, for the final Multiple Clustering Analysis. It is important to consider that each clustering solution in an MCA is derived by the quantitative measurements alone, in an unsupervised manner. However, biological enrichment is used to shape the final MCA representing a feature selection step in the MCAM method. 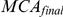 is typically on the order of 500 clustering sets. With this final MCA we can begin to compile the biological information that is contained, explore how clustering parameters compare to each other using mutual information, how clustering parameters relate to particular biological metrics, and consider biological hypotheses generated throughout all of these steps.

**Table 1:** Description and categorization of biological metrics.

Metric Level	Metric	Short Name
Protein Metrics	Gene Ontology: Molecular Function	F
	Gene Ontology: Biological Process	P
	Gene Ontology: Cellular Compartment	C
Site Metrics	Pfam Domains	Pfam
	Domain Phosphorylation	Pfam Site
	PhosphoELM Kinase Annotations	PELM Kinase
	Linear sequences	Motifs
	Scansite Kinase Predictions	Scansite Kinase
	Scansite Binding Predictions	Scansite Bind
Dynamic Quantitative Metrics	Minimum Phosphorylation	MinValue
	Maximum Phosphorylation	MaxValue
	Maximum Positive Change	MaxPosChange
	Maximum Negative Change	MaxNegChange

There are a total of nine biological metrics and four dynamic metrics analyzed for enrichment in clusters. The metrics tested describe information regarding the protein a peptide arises from, the particular site of phosphorylation, or the quantitative data. The abbreviated labels used throughout this work are given.

In this work we will focus on studies of ERBB network dynamics. In a first example, we evaluate a four time point measurement of the 184A1 human mammary epithelial cell (HMEC) line stimulated with a saturating concentration of EGF, where measurements were taken before stimulation (0 min) and then subsequently at 5, 10 and 30 minutes following EGF addition [26]. Enrichment and fractionation steps focused on capturing tyrosine phosphorylation signaling events in the ERBB network. This dataset represents extensive measurement of the phosphotyrosine ERBB signaling network, with 77 unique phosphopeptide measurements on 68 proteins. Throughout this work we will refer to this dataset as *EGF4* for brevity. This dataset, a 77×4 matrix, represents the relative quantitative measurements of 77 phosphopeptides in time following EGF stimulation, where the vectors used in clustering are the dynamic measurements of a single phosphopeptide. The full dataset is plotted in principal component space in Supplementary Figure S1. The clustering parameters originally applied, and those removed during pruning, are described in Table 2. The original MCA included 1,320 sets and after pruning, 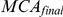 included 331 total sets. Pruning indicates that for this dataset and set of parameters, the use of Hierarchical clustering, set sizes of two and four, the differential transformation, and the use of correlation and cosine as a distance metrics are uninformative in producing enrichment in the categories tested. The 331 sets remaining after parameter refinement produces a wide diversity of biological enrichment across all categories of information.

**Table 2:** Clustering Parameters and MCA Set Sizes.

Parameter Type	Original Set	Pruned
K	2, 4, 6, 8, 10, 12, 14	2, 4
Transform	raw (untransformed)	
	center (zero centered)	
	zscore (mean center, sample standard deviation of 1)	
	normMax (normalized to maximum value)	
	rangeScale (full range scaled to 1)	
	log10 (log base 10)	
	pow (power to 0.5)	
	pareto	
	FFT	
	diff (differential)	diff
	normMax_log10 (normMax follwed by log10)	
	zscore_log10 (zscore followed by log10)	zscore_log10
Distance	euclidean	
	correlation	correlation
	cityblock	
	cosine	cosine
	chebychev	
Algorithm	Ncut (Ncut Segmentation)	
	AP (Affinity Propagation [18])	
	SOM (Self-Organized Map [25])	
	Kmeans	
	Hierarchical	Hierarchical
Set Size	1320	331

The parameters of clustering used in application to the *EGF4* dataset including the short names used throughout this work as well as increased description of the parameters. Pruned parameters are parameter sets removed from 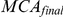 because their removal improved overall biological enrichment by at least 2% and did not negatively affect any one category by more than 10%.

### Validation of biological enrichment

**Figure 2:**
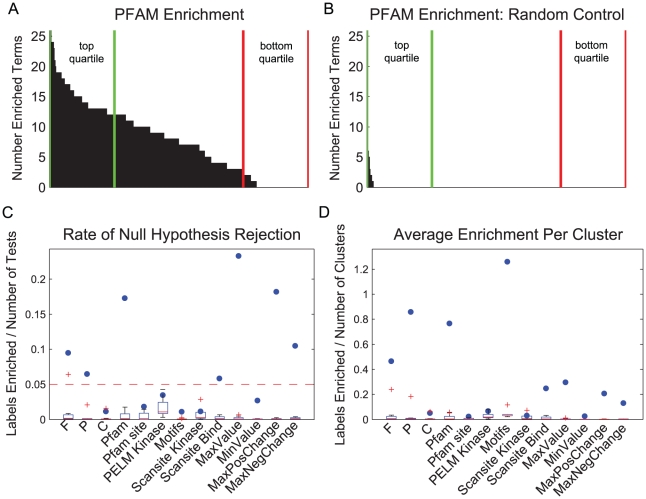
Biological enrichment in MCA compared to random controls. A) An example histogram of PFAM enrichment in the MCA plotted in descending order of the number of total terms enriched per set. Green lines mark the top 25% and red the bottom 25% of sets based on total number of labels enriched. B) Example resulting enrichment from a random control for PFAM enrichment. C) The rate of null hypothesis rejection, per biological category, for 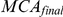 and ten random controls. Random control distribution plotted in whisker plot and blue circles represent MCA results. Null hypothesis rejection in a random control is equivalent to a false positive, which as controlled for using the False Discovery Rate procedure with a cutoff of 0.05. D) Resulting average enrichment found, per category, per cluster in 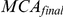 (blue dots) and ten random controls (box plots).

In order to ensure that the basis of our analysis for clustering fitness is a product of actual biological power, versus the production of a large degree of false positives due to Type I error, we performed ten random controls, Figure 2 and Supplementary Figure S2, using two different randomization methods. In the first method, the data matrix is randomly reshuffled and subjected to clustering using the same parameters as in 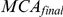, followed by enrichment analysis. This method should control for the mechanics of clustering random data. The second method randomized the biological labels assigned to the vector dynamics by randomly reshuffling the assignment of phosphopeptides to the measured phosphorylation dynamics. This randomization process should control for the potential limitations in the annotations used for enrichment. Both methods gave similar results, and if not explicitly stated otherwise, the results described in this paper refer to randomization using the first method described.

Figures 2A and B depict the extent of biological enrichment found in one representative metric, PFAM domains, for both real data and a representative random control. Figures 2A and B represent the total number of enriched PFAM labels found per cluster set, in order of descending degree of enrichment per set. The ‘top’ and ‘bottom’ quartiles, according to total enrichment per set, are indicated since they will be used extensively throughout this work. As expected, we do see some enrichment in the random control, which represents a small number of false positives, however the contrast between the results of clustering real biological data and random data is stark. These observations indicate that clustering of temporal phosphorylation measurements is capable of producing meaningful biological enrichment and may therefore be useful for inferring function and regulation of poorly understood phosphorylation events in the ERBB network.

To ensure that Type I error, or the false positive rate, is controlled, at least empirically, at the target rate of 0.05 or better, we plotted the rate of null hypothesis rejection across all metrics for real data and for the 10 random controls, Figure 2C. Figure 2C shows that for most categories the rate of null hypothesis rejection is higher than the random controls, and for all controls Type I error is empirically controlled at 0.05 or better, with only one outlier in the case of GO Molecular Function terms. We also wanted to explore the total number of statistically significant labels produced in real versus random MCA sets, shown in Figure 2D and Supplementary Figure S2 for randomization using the second method described above. Bonferroni correction was also used and the results were similar regarding empirical control of the false positive rate, but demonstrated a large loss in statistical power, Supplementary Figure S3. Given these results, FDR correction is used in all further results presented.

Figure 2 shows some categories, like motifs, which have roughly the same rate of null hypothesis rejection as random controls, have a much larger number of total hypothesis tests, resulting in a large degree of enrichment in the case of real biological data. However, some categories perform no better than random controls based on both rate of null hypothesis rejection and total enrichment. These categories include Scansite Kinase predictions, PhosphoELM kinase annotations, and phosphorylation within known PFAM domains (pfam_site). We noticed that despite the fact that overall enrichment for Scansite Kinase terms was poor in 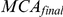, clusters derived using the Fast Fourier Transform, FFT, were correlated with production of Scansite Kinase enrichment in a significant manner. We looked at random controls and real enrichment for Scansite Kinase terms in just FFT derived clusters and found there was an appreciable improvement in enrichment for this relative to random controls Supplementary Figure S4, indicating that for specific enrichment, subsets of 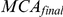 could be considered. This presents a method in which to target clustering results for producing meaningful information in a particular biological category. For example, here it appears that the FFT subset, which performs better than random controls for producing Scansite Kinase predictions, may provide clusters of phosphorylation sites with the power of inferring shared kinase regulation. Taken together, these results demonstrate that the use of any single algorithm, distance metric, and transformation would highlight only a small fraction of the possibly interesting relationships between the data.

### Analysis of clustering parameters

We originally hypothesized that the choice of optimal unsupervised learning parameters would be dependent on the biological information desired in the resulting clustering solution. Therefore, we explored the relationship between clustering parameters and the enriched biological information. We rank ordered the sets in an MCA in nine ways, according to the degree of total enrichment the sets contained within each of the nine biological metrics, depicted in Figure 2A for PFAM domain ranking. We found that there was no single set that performed in the top 25% of all nine metric rankings. When we took the union of the top 25% in the nine orders, we found that 273 sets of the 331 sets in the MCA, or 83% of all sets, were required to capture the top quartile of all biological information. These results indicate that, indeed, the choice of ‘optimal’ parameters will be based on the desired type of categorical relationships, since almost all sets are required to capture the full array of possible information.

To understand how parameters directly relate to specific types of biological information, we tested for the overrepresentation of clustering parameters in the top and bottom quartiles of all nine biological metrics, and four dynamic metrics, Table 3. We found that the parameter performance is based on the particular metric tested. For example, the cityblock distance metric consistently performs poorly for producing clustering sets with clusters that have enrichment in GO Molecular Function terms, but the same distance metric consistently performs well in producing sets with clusters containing PFAM enrichment. Interestingly, Kmeans clustering is in the bottom quartile of all biological metrics. However, since Kmeans was not cut in the reduction stage of creating 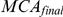, it indicates that is still useful at producing biological enrichment, unlike those parameters shown in Table 2 that were pruned based on their inability to produce significant biological meaning. The lack of enrichment for a parameter in a particular category indicates that the top and bottom quartiles have diversity of parameters in this category. For example, there are transforms, log10 and normMax, that consistently produce clusters with motif labels, but there is no particular distance metric or algorithm that outperforms others in this capacity. This observations indicate that there is indeed an important relationship between the parameters chosen in clustering and the resulting biological information produced.

**Table 3:** Relationships between parameters of clustering and biological enrichment.

Metric		Top Quartile	Bottom Quartile
F	K		6
	transform	FFT, center	
	distance	chebychev	cityblock
	algorithm		Kmeans
C	K		
	transform	FFT,center	
	distance	euclidean	cityblock
	algorithm	SOM	Kmeans
P	K		6, 8
	transform	FFT, center	log10, nMaxLog10, rangeScale
	distance		
	algorithm	AP	Kmeans
PFAM	K	12, 14	
	transform	pow	zscore, rangeScale
	distance	cityblock	
	algorithm		Kmeans, SOM
Motifs	K	6	10
	transform	log10, normMax	zscore
	distance		
	algorithm	Ncut	
Scansite Bind	K	6	14
	transform	nMaxLog10	
	distance		
	algorithm		Kmeans, Ncut
Scansite Kinase	K	12	6
	transform	FFT	
	distance		
	algorithm		

Parameters are given for each biological metric if they are enriched in either the top or bottom quartile of the list when ranked by the number of labels enriched in that category. PELM kinase annotations and Pfam_site did not perform better than random controls, and are not included. Although Scansite Kinase parameter enrichment also did not perform better than random controls, it is listed here since subsets based on the FFT did perform better than their random control counterparts. See Table 2 for a full description of parameters.

**Figure 3:**
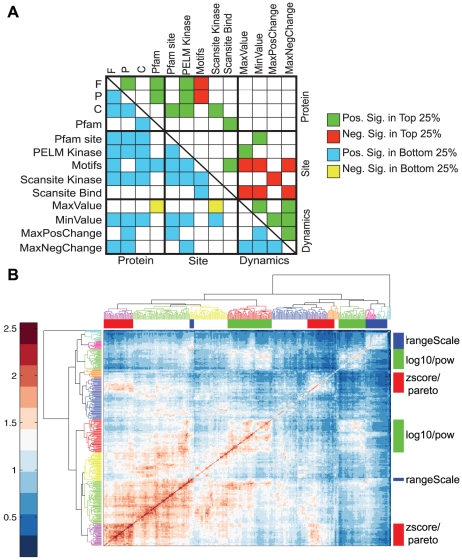
Comparison of parameters and metrics. A) Pairwise comparison of the overlap in the best and worst 25% of sets based on each metric in 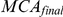. We performed 1000 random selections of two sets of the same size to generate a normal distribution whose mean represents the expected overlap value between any two sets pulled from that background size. We then evaluated whether pairwise overlap was significantly higher (‘Pos. Sig’) or lower (‘Neg. Sig.’) than expected by random. Significance cutoff was set at a FDR corrected alpha value of 0.05. The top right represents the pairwise comparison of the best performing 25% and the bottom left is the comparison of the worst performing 25% of sets in each category. B) Hierarchical clustering of pairwise mutual information between every set in the MCA. Self-MI is highest along the diagonal. Highlighted groups indicate dendrogram cutoffs for which the full group is composed of the denoted parameter. The labels log10/pow denote normMax_log10, log10 and the pow transformations, pareto/zscore contain zscore and pareto transformations. The topmost zscore/pareto group contains one outlier (out of the group of 41) created using the transform pow.

We discerned additional results in Table 3 to be worth exploring further. For example, it seems that the FFT transformation performs well in producing multiple protein-level metric types. To elucidate this, we compared the set rankings in all 13 metrics (biological and dynamic) in a pair-wise fashion. Using a bootstrapping method, we compared the overlap in the top and bottom quartile rankings with that expected by random and highlighted when there was either a significant increase or a significant decrease between any two metric rankings compared to the expected overlap, Figure 3A. The most striking results from this analysis are that there tends to be relatively good agreement in the top and bottom quartiles of sets between protein-level information and site-level information, with the exception of motifs. However, there is very little overlap between different site-level categories in the top quartile, except for Scansite Binding predictions and motifs. There are roughly twice as many positively enriched overlaps in the bottom quartile than there are the top quartile indicating there tends to be more agreement in the worst performing sets across different categories than in the best performing sets. Enrichment for dynamic terms is meant to act as a marker for those features in the dynamics that may have led to a particular clustering outcome. There is a good degree of agreement between dynamic enrichment, but very little agreement with protein-level information and opposing agreement with site-level information.

In order to compare how the phosphopeptide cluster mappings between different clustering solutions compare, we calculated the mutual information between sets in a pairwise fashion. The pairwise MI calculations have been hierarchically clustered in Figure 3B, high values of MI indicate a closer agreement in two clustering solutions than low values of MI. The clustered heat map of MI terms indicates there are pockets of sets with high agreement. We bisected the MI cluster tree at various levels and searched for indications that a set of parameters drives similarity between set architectures. At the coarsest level, and depicted in Figure 3C, similarity appears to be driven by the data transformation. At the next level, it appears the algorithm defines similarity and finally the distance metric. There was no clear evidence that cluster set size, K, was important in determining similarity, but this is a poor method for determining the exact effect of K, since the maximum possible MI between any two sets, or self-MI, will be dependent on their cluster set sizes K. We took subsets of 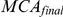, based on having a single shared parameter, such as all those sets derived with a log10 transform, and found the average MI in that subset. The distributions, Supplementary Table S1, indicate that there is far more similarity in the clustering set solutions when single transform subsets are explored, than when single algorithm or distance metric subsets are explored, which is in agreement with the observations from Figure 3C. Using this same subset MI averaging method, we can look at direct comparison of the similarity of any two parameters of clustering by creating a subset made up of both parameters, Supplementary Table S1. Rank-ordered lists for data transformations also agree with observations from Figure 3B, for example, that log10 transform clustering solutions are most related to the power transform solutions and least related to rangeScale transform solutions. Additionally, we find that Kmeans and AP clustering algorithms perform the most similarly to each other. However, both Ncut and SOM algorithm subsets show a higher similarity with other algorithms than they do with themselves. These observations could be very useful in determining a subset of clustering parameters that could be chosen to generate the maximum difference in clustering solutions.

### Biological inference capabilities

In the previous section, we developed methods for comparisons of parameter and metric information in order to understand the impact parameters have on biological enrichment, the similarities between biological metric categories, and the impact parameters have on clustering solution architectures. In addition to this, we wanted to develop a way to use the resulting MCAM information in a more traditional manner, regarding biological inference and hypothesis generation. There are a variety of ways in which one could select a manageable number of clustering solutions from 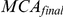 for manual evaluation based on selecting the sets with the largest differences in architecture or resulting biological information. However, here we decided to look at robust relationships that result despite the high degree of variability that occurs through the implementation of a combinatorial set of clustering parameters. We did this in two ways, first by combining the enrichment results of 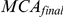 and second by looking at the frequency of which any two phosphopeptides co-cluster in 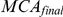.

**Figure 4:**
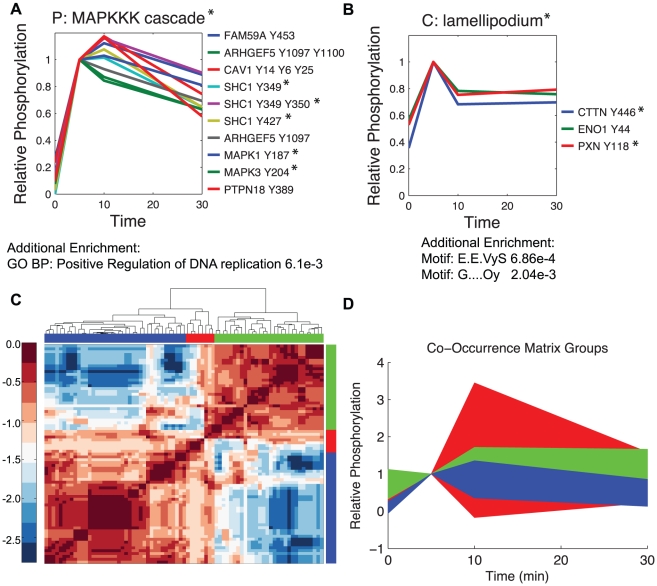
Biological inference based on robust clustering results. A) The group of phosphopeptides that participate at least 50% of the time in a cluster with enrichment for GO Biological Process term “MAPKKK Cascade”, those proteins with the term are starred. This new group is enriched for GO BP term “positive regulation of DNA replication”. B) These three phosphopeptides always appear when GO Cellular Compartment term “lamellipodium” is enriched, CTTN and PXN are the proteins annotated as being localized in lamellipodium. This new group is enriched for two sequence motifs as well. C) The co-occurrence matrix clustered hieararchically. Co-occurrence between any two phosphopeptides is the number of times those two peptides are clustered together in 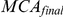. For the heat map, the log base 10 was taken of the normalized values, zero values became 0.5/331 prior to log transformation. D) The average values, +/− two standard deviations, are shaded for the three groups highlighted in panel C.

Supplementary Table S2 lists all biological and dynamic enrichment that occurs in 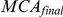 and the number of times that label occurs. Across the biological categories of interest, there are 539 unique labels enriched at least once in 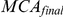, and many of these labels appear in more than one clusterset. A histogram of the number of unique biological labels enriched in 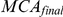 versus the number of clustersets they occur in is given in Supplementary Figure S5. Biological labels enriched several times are considered to be ‘robust’ labels, for example GO Biological Process terms “DNA binding” and “transcription factor binding” both appear in 23% of the cluster sets. Since these robust labels may contain specific information worth exploring, we seeded the generation of a “robust cluster” based on the number of times phosphopeptides participate in a cluster with a particular enrichment term. Figure 4A and B show the results of two such terms, GO Biological Process term “MAPKKK cascade” and Cellular Compartment term “lamellipodium” and all phosphopeptides that were in a cluster giving rise to these terms at least 50% of the time. In addition to being enriched for the seed term, Figure 4A, this ‘robust’ cluster is also enriched for GO:BP term “positive regulation of DNA” proliferation. This group is composed of multiple phosphopeptides from SHC1 (Swissprot P29353), an adaptor protein recruited to the EGFR in response to EGF and upstream of the MAPK cascade. Additionally, it includes the activation sites of MAPK1 (ERK2, Swissprot Q1HBJ4) and MAPK3 (ERK1, Swissprot Q7Z3H5) and a phosphorylation on a relatively poorly characterized protein, FAM59A (Swissprot Q9H706). A recent study showed FAM59A acts in the MAPK pathway in response to EGF stimulation by binding to Grb2 in a manner that is dependent on the phosphorylation of Y453 [27]. The group associated with the cellular compartment “lamellipodium” is composed of only three phosphopeptides from the proteins cortactin (CTTN, Swissprot Q14247), paxillin (PXN, Swissprot P49023), and ENO1 (Swissprot Q96GV1), an enolase. Paxillin and cortactin are both labeled as being localized in lamellipodium, however, ENO1, at least in full length is currently thought to be only cytoplasmic. This robust “lamellipodium” cluster also has enrichment for two sequence motifs, E.E.VyS, which is shared in both PXN and CTTN and G....Oy (O indicates a degenerate search for hydrophobic amino acids and ‘.’ for any amino acid), common to ENO1 and CTTN. These motifs may be indications of shared enzyme or binding domain recognition. These findings indicate that productive biological inference regarding function, localization and regulation is possible using the MCAM framework.

An alternate method for understanding relationships between data measurements is to consider the frequency that any two phosphopeptides appear in the same cluster. We calculate this frequency, or co-occurrence, for every pairwise combination. The hierarchically clustered co-occurrence matrix is shown in Figure 4C. It appears in Figure 4C that there are essentially two groups of peptides that co-cluster a great deal with themselves and very little with the other group, listed explicitly in Supplementary Table S3. We highlighted these two groups, and one we consider a ‘transition’ group and plot the average dynamics of those groups +/− two standard deviations in Figure 4D. The transition group clearly has the largest variability in their dynamics, which may explain why they co-cluster to some extent with both outlying groups. The “blue group”, which is also the largest, appears to have the least variance in the early time points following EGF stimulation. There is also a marked difference between the downregulation of the two outlying groups. Analysis of the co-occurrence matrix indicated there are roughly only two main dynamics in tyrosine phosphorylation dynamics within this dataset. However, given that enrichment analysis was very fruitful for finer gradations, it is clear that several levels of dataset separation are useful.

With 77 phosphopeptides, there are 2,926 pairs of phosphopeptides to consider in the *EGF4* dataset, listed in Supplementary Table S3. In 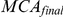, only 63, or 2.15%, of the possible relationships never occur. Fifty-percent of the possible relationships occur less than 34 times. There are 272 pairwise clusterings that occur more than half the time in 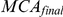, which accounts for 9.3% of possible relationships. We found no particular relationship between the parameters of clustering and the production of infrequently, or highly frequently, occurring phosphopeptide clustering. The most robust relationship seen is STAT3 Y705 (isoform 1, Swissprot P40763) phosphorylation with STAT3 Y704 (isoform 2, Refseq NP_003141) phosphorylation, co-clustering all but two times. This variability highlights the importance of considering more than a single clustering solution when deriving hypotheses for further testing.

We have illustrated only a few examples of a large number of possibilities to demonstrate the power of MCAM in deriving biologically meaningful hypotheses. We encourage others to make full use of the Supplementary Information and Matlab scripts to continue to explore the results in this dataset. In particular, the methods that are the most promising, which are described in this section, are to explore robust relationships found either: 1) Through the exploration of a particular enriched label of interest, or 2) Through the exploration of a particular phosphopeptide of interest and those phosphopeptides that co-cluster the most robustly with it.

### Comparison of multiple ERBB datasets

We wished to explore the utility of MCAM in comparing independent measurements of the same network, and so we turned to a different study of ERBB network dynamics by Wolf-Yadlin et. al. [12]. In this study, which will be referred to here as the *HER2* dataset, the authors were interested in the signaling response downstream of EGF and HRG and in response to HER2 amplification, which is common to several breast cancers. They used two cell lines, the wild type, or parental HMEC cell line, which is the same cell line used in the *EGF4* study described previously in this paper, which has 20,000 HER2 receptors per cell, and 24H, a HER2 overexpressing cell line, which has 600,000 HER2 receptors per cell. Both cell lines express roughly the same number of EGFR and HER3 receptors. EGF ligand binds EGFR, which will drive EGFR homodimers and EGFR:HER2 heterodimers, whereas heregulin, HRG, which only binds the HER3 and HER4 family members will instead drive HER3 and HER4 containing heterodimers. When HER2 is overexpressed, the majority of the dimers in HRG treatment will be composed of HER2:HER3 and a larger proportion of EGFR:HER2 dimers will occur in response to EGF treatment. The authors found HER2 overexpressing cells were more migratory in response to stimulation by either ligand when compared to wild type HMECs. One quarter of this dataset represents the same measurement conditions as the *EGF4* dataset (0, 5, 10, and 30 minutes following a saturating dose of EGF) of wild type HMECs (termed P for parental). The remainder of the dataset is the measurement, at the same time points, of parental with HRG treatment and with HER2 overexpressing (24H) cells treated with EGF and HRG. We were interested in applying MCAM to the *HER2* dataset in order to see if two independent measurements of a network would agree and if signaling differences between EGF and HRG stimulation, and wild-type versus HER2 amplification, could be distinguished using MCAM and could therefore highlight potential signaling mechanisms related to the increased migratory behavior of HER2 overexpressing cells. We applied MCAM to the *HER2* dataset and to five subsets of the dataset in order to tease apart: 1) differences in signaling between the various HER2 states and ligand treatments, and 2) how measurements of a conditional nature (for example measurements at only one time point across the four conditions) would differ from the dynamic measurements and how these differ from the full dataset. The datasets analyzed by MCAM include: the full 16-point measurement set (Full), a “conditional” dataset made up of the 5-minute measurements in all conditions (5 minTimePoints), and four subsets representing the four dynamic measurements (0, 5, 10, and 30 minutes) of each condition named P_EGF for EGF stimulation of Parental cells, P_HRG for HRG stimulation of parental cells, 24H_EGF for EGF stimulation of HER2 overexpressing cells, and 24H_HRG for HRG stimulation of HER2 overexpressing cells.

To understand more globally how the MCAM results compare across these different datasets, we found the correlation between the co-occurrence matrixes. The MCAM co-occurrence matrix of the *EGF4* dataset has the highest correlation with that of the Parental EGF subset of the *HER2* dataset, followed closely by the EGF treatment of the 24H cell line, there are 36 phosphopeptides common to both the *EGF4* and *HER2* datasets, Table 4. There is poorer correlation with the HRG treated subsets and the worst correlation with 5 minuteTimePoint subset. In general, we see that EGF and HRG treatments correlate much better with themselves than they do with each other. For example, all EGF treatment comparisons appear in the top of the rankings of correlations, with an average correlation of 0.46. However, all cross-treatment comparisons have correlation of 0.35 or worse, with an average correlation of only 0.25. Using the co-occurrence matrix that results from MCAM application to different measurements of the same system is a useful way to derive a single, global metric for the agreement between different datasets.

**Table 4:** MCAM co-occurrence correlations of ERBB network datasets.

Dataset 1	Dataset 2	Correlation	Ligands
Full	5 minTimePoints	0.63	
Full	24H EGF	0.59	
EGF4	P EGF	0.53	EGF:EGF
EGF4	24H EGF	0.51	EGF:EGF
Full	24H HRG	0.45	
Full	P HRG	0.39	
EGF4	Full	0.37	
P EGF	24H EGF	0.36	EGF:EGF
P HRG	24H HRG	0.35	HRG:HRG
EGF4	P HRG	0.35	EGF:HRG
5 minTimePoints	24H HRG	0.34	
Full	P EGF	0.32	
5 minTimePoints	24H EGF	0.31	
EGF4	24H HRG	0.25	EGF:HRG
24H EGF	24H HRG	0.24	EGF:HRG
5 minTimePoints	P HRG	0.23	
P EGF	P HRG	0.23	EGF:HRG
P HRG	24H EGF	0.23	EGF:HRG
P EGF	24H HRG	0.17	EGF:HRG
EGF4	5 minTimePoints	0.16	
5 minTimePoints	P EGF	0.10	

The co-occurrence matrix correlations for the MCAM results of *EGF4* and six datasets obtained from the full *HER2* dataset were found for those phosphopeptides contained in both Dataset 1 and Dataset 2. The lists are rank-ordered according to decreasing correlation and ligands are highlighted when a dataset is composed only of that treatment condition. The line separates EGF:EGF and HRG:HRG comparisons from the EGF:HRG comparisons. The correlation of *EGF4* with subsets of the *HER2* dataset involves 36 common peptides and all *HER2* comparisons involve 68 phosphopeptides.

**Figure 5:**
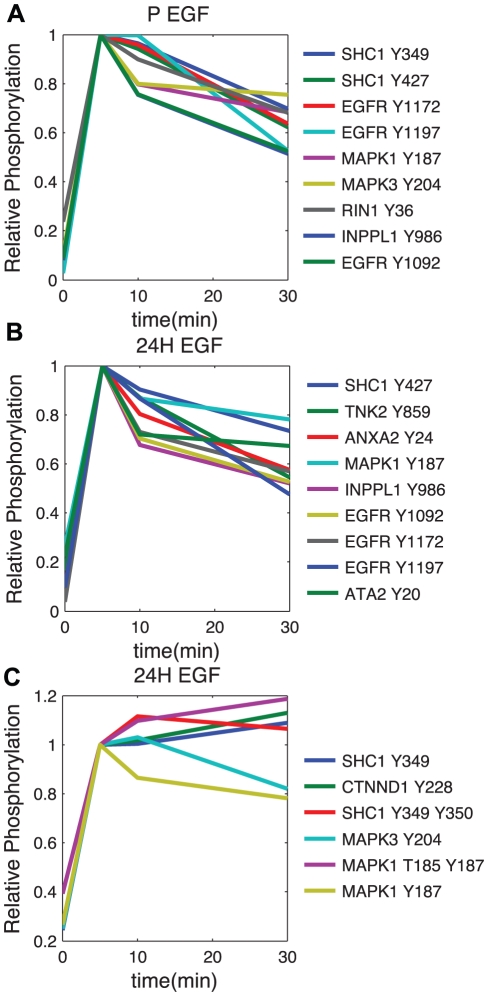
Dynamics for SHC phosphorylations and their robust co-clustered partners in EGF treatment of parental and HER2 overexpressing (24H) cells. For these plots, all subsets were normalized to their own 5 minute time point to make the comparisons across treatments easier. Peptide centric clusters were created by finding all peptides that co-cluster with a given peptide at least 50% of the time in 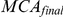. A) Both SHC1 Y349 and SHC1 Y427 centric clusters are the same for Parental HMEC cells treated with EGF. B) EGF treatment of 24H cells creates some change in the SHC1 Y427 centric cluster, but the dynamics compared to Parental treatment are relatively similar. C) The SHC1 Y349 centric cluster has changed drastically compared to Parental EGF treatment due to a very different response of Y349 phosphorylation.

In order to explore the differences in clustering results of any two datasets, we are faced with analyzing an overwhelming number of comparisons. To simplify the search range, and highlight those differences that are the most extreme, and potentially the most biologically interesting, we looked for co-occurrences that move from one extreme of co-clustering to another. Specifically, we define an extreme difference as the case when two phosphopeptides change from co-clustering at least 75% of the time in one dataset to co-clustering less than 25% of the time in another. Full results for all dataset comparisons are provided in Supplementary Table S4. There are several notable changes in cross-comparing the *EGF4* dataset and all sets formed from the *HER2* dataset, but the ones we found the most interesting occur in the difference between EGF treatment of parental cells versus 24H cells. In this comparison, we noticed two sites on SHC1 experience very different phenomena. In the case of parental EGF treatment, both sites co-cluster robustly with each other and with phosphorylation on EGFR pY1172 and pY1197 (Swissprot P00533), both known to bind SHC1. However, EGF treatment of HER2 overexpressing cells indicates that although pY427 continues to robustly co-cluster with those sites on EGFR, SHC1 pY349 does not, and instead most robustly co-clusters with catenin delta-1, CTTND1 (Swissprot O60716), phosphorylation of Y228. Dynamics of these sites, and their most robustly co-clustered partners, under both conditions are shown in Figure 5. We also observe large differences in the co-clustering of multiple sites on p130Cas, also known as BCAR1 (Swissprot P56945), between the two cell lines in response to EGF treatment and an extreme change in the association of ENO1 pY44 with annexin A2 (ANXA2, Swissprot P07355) phosphorylation on Y238, where ENO1 Y44 phosphorylation associates with ANXA2 phosphorylation in parental but not HER2 overexpressing cells. The largest number of differences is observed when comparing the EGF treatment of parental cells to the 24H cell lines treated with HRG, which also has the lowest correlation of any dynamic subset comparison. Both pieces of evidence point to maximum differences in signaling dynamics when both HER2 expression levels and the stimulating ligands are altered. Further hypothesis generation can be accomplished by exploring the remaining pairwise dataset comparisons for other meaningful signaling changes that occur, which are highlighted by extreme differences in phosphopeptide co-clustering, provided in Supplementary Table S4.

Although the best agreement the *EGF4* MCAM results have is with the parental EGF treatment of the *HER2* MCAM results, there is less than ideal correlation. We looked to see if there were any serious disagreements by using the same ‘co-cluster swap’ method as mentioned above and found there were only two such instances, which highlight very different measurements made on GIT1 (Swissprot Q9Y2X7) pY545 and EFNB2 (Swissprot P52799) pY304 in HMEC cells in response to EGF treatment, Supplementary Table S4. Interestingly, though these measurements are quite different, the extreme differences of co-occurrences with other phosphopeptides are only highlighted in two cases, both with regards to Ephrin family phosphorylation sites that are relatively similar in the two datasets, GIT1 pY545 with EPHB1 (Swissprot P54762) pY600 and EFNB2 pY304 with EPHA2 pY772 (Swissprot Q96HF4). This indicates that the MCAM co-occurrence matrix is a precise way to identify similarities and discrepancies between independent measurements of a system. Since this methodology relies on comparing the relationships between measurements within a dataset and then comparing this abstracted metric across datasets, one could imagine that the measurement scheme would not need to be the same between the two datasets of interest.

## Discussion

MCAM was developed to capitalize on the success unsupervised learning has had on biological inference in the past and apply it to a new challenge in the field, that of understanding the function and regulation of phosphorylation in the ERBB network. Since the dynamic trajectory of a phosphorylation site will be shaped by the combination of the kinase and phosphatase activities, as well as any protective influence accumulated through binding to other proteins, and the exposure to all of these proteins by localization with or away from their regulatory proteins, then co-regulation of multiple phosphorylation sites may yield testable hypotheses regarding one or all of these possible shared traits. Encouragingly, biological information is enriched in dataset partitions derived through separation by clustering, whereas multiple random controls do not demonstrate a significant relationship with biological category enrichment. In addition to using this information to gain understanding of the phosphorylation events in the ERBB network, we also undertook to account for the fact that a large number of mathematically correct solutions can be derived through the use of various data transformations, distance metrics, algorithms, and target set sizes. We found that given a variety of clustering solutions, one could see almost any pair of phosphopeptides co-cluster, which may not necessarily be a product of co-regulation that can be linked directly to spatial localization, shared functionality, or shared enzymatic regulation. Therefore, we feel any single clustering implementation will have hypotheses likely to yield testable and supportable information as well as a good mix of those that may not.

Each clustering set within an MCA represents the dimensionality reduction of a large, multidimensional dataset. However, we found there is unique value in the majority of all clustering sets in 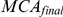, according to the variety of biological enrichment, and so no single set could be considered as an optimal solution and evaluated in a traditional way. Therefore, the most significant development that had to be made in MCAM was to reduce the complexity of a large number of clustering sets. To this end we focused on two aspects of information: 1) Understanding how the parameters affected the final clustering solution, and how this related the power of any particular type of biological inference that could be made, based on statistically enriched information and 2) How to derive meaningful and testable biological hypotheses, through inference, concerning the function and regulation of protein phosphorylation. We found the resulting methods provided important insights when comparing similar measurements, indirectly, across multiple datasets.

Through the use of this technique we found that indeed, optimal clustering parameters one would choose for clustering a dataset would vary greatly dependent on the type of information that was desired at the outcome. However, for those biological categories chosen in this study, there were a few parameters that performed badly across the board, including Hierarchical clustering and the differential transform. These are somewhat unsurprising since the differential transform will reduce the vector size by one dimension, which in a dataset with few measurements may be detrimental to the ability to separate the measurements. However, it is an important observation to mention that lessons found in one dataset should not be extrapolated to all datasets, even of the same type. Although the cosine and correlation distance metrics were uninformative in producing biological enrichment in the *EGF4* dataset, they were useful in clustering various portions of the *HER2* dataset. This observation highlights the importance of a broad-spectrum application of multiple parameters of clustering.

It is important to consider the impact the feature selection process has on the final result in the MCAM method, which is applied during the parameter refinement, or pruning, step of the MCAM method. Given the varied relationships we observed between particular types of information and the parameters of clustering that best gave rise to them, it is likely that the final result will change with the addition of new biological features, or the alteration to existing features, such as the improvement of GO annotations. One can imagine the parameters pruned during the feature selection process would decrease with the addition of new features, which would redefine the value of a successful clustering solution. Although this suggests the results may need to be reconsidered as annotations improve, we feel this parameter refinement process helps to avoid the consideration of a large portion of currently uninformative sets. The open source nature of the MCAM software project allows for flexibility in altering the specific categories and the thresholds used during parameter refinement.

We found that there is no single ‘optimum’ clustering solution, or one that performed in the top quartile of all biological metrics of interest. Depending on the application, a small number of solutions could be chosen and analyzed in a way that is more traditionally performed in the field. However, we decided to focus on allowing the agreement of many solutions to highlight a potential area of robust biological inference through the agreement of biological enrichment and alternately the agreement of co-clustering of phosphorylation dynamics. These methods produced a large list of possibly interesting biological inferences, of which we highlighted just a few possibilities to demonstrate MCAM's power. In Figure 4 we highlight two ‘robust’ clusters based on repeated enrichment of categorical terms, which creates a hybrid cluster from a combination of multiple clustering sets within 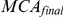, based on a particular enrichment label of interest. The hybrid cluster, like any single cluster produced from a single clustering method, represents a cluster of phosphopeptides that are strongly co-regulated. The first cluster, Figure 4A, was produced based on enrichment for co-regulation of phosphorylation sites on proteins involved in the MAPK cascade. For those proteins not currently annotated in the MAPK cascade, there is individual evidence that they are involved in regulating MAPK activity. FAM59A was recently named GAREM, which stands for Grb2-associated and regulator of Erk/MAPK activity [27]. Specifically, phosphorylation of Y453 on GAREM was required for association with Grb2 and subsequent activation of Erk by EGF stimulation. PTPN18, a protein tyrosine phosphatase also known as BDP1, has been implicated in regulation of HER2 directed MAPK signaling activation [28]. The study specifically found that PTPN18 was capable of inhibiting activation of mitogenic signaling. The robust co-regulation of PTPN18 Y389 phosphorylation with other components of the MAPK cascade, shown here, further implicates PTPN18 in MAPK signaling downstream of EGF stimulation, and highlights a particular mechanism for PTPN18 activity, that of Y389 phosphorylation. This modification may possibly act as a negative regulator of BDP1 activity, thereby relieving its function as a negative regulator of MAPK activity. Alternatively, if Y389 phosphorylation on PTPN18 potentiates its ability to shut down MAPK signaling, then these dynamics suggest it occurs subsequently with MAPK activation. ARHGEF5, also known as TIM, is a RhoGEF, which has been shown to activate Rac, which is upstream of another MAPK family member, JNK. This suggests that the JNK cascade may be concurrently activated or that this particular RhoGEF has a role in the ERK1/ERK2 cascade directly. These results indicate that MCAM has been useful not only in highlighting a known co-regulation event, that of EGFR phosphorylation on sites that recruit SHC1 to the receptor with that of SHC1 phosphorylation sites that are phosphorylated following recruitment to the receptor, but also in highlighting proteins not yet generally recognized as playing a role in the EGFR/MAPK signaling pathway. This result also supports a role for these proteins in the MAPK pathway in human mammary epithelial cells as previous cell lines explored in previous studies. Additionally, the example of highlighting GAREM (FAM59A) phosphorylation on Y453 as playing an important biochemical role in the indicated pathway strengthens the hypothesis that PTPN18 Y389 phosphorylation is also an important biochemical mechanism in the MAPK pathway downstream of EGFR activation. Since we can find no specific study on the effect of PTPN18 Y389 phosphorylation, this hypothesis could not have come from literature mining. Additionally, we observe that traditional application of a single clustering implementation would likely not have highlighted this group of proteins in a way that would have linked PTPN18 Y389 and GAREM Y453 phosphorylation with that of EGFR Y1172 and MAPK Y187, since this event occurs in less than 15% of clustering set solutions in 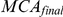.

In Figure 4B we highlight another ‘robust’ cluster, which indicates the dynamics of phosphorylation of Y44 on ENO1 are very similar to that of cortactin and paxillin phosphorylation, two proteins and phosphorylation sites that play a role in cell motility [29], [30] and are annotated as being localized to ‘lamellipodium’. However, since there is relatively little evidence that paxillin is localized to lamellipodia, but instead has a strong association with mature focal adhesions [29], it may be that ‘lamellipodium’ has been used as a blanket term for leading edge formations in the Gene Ontology since GO is lacking finer gradations of leading edge compartments. What is common to both paxillin and cortactin is localization in invadopodia [31], [32], a term not currently included in GO. Such similarity of dynamics indicates potential co-regulation, which is dependent on a variety of factors. There is evidence that all three sites may be targets of Src [30], [33], [34]. In addition to shared enzymatic control, similarity of dynamics might also be dependent on shared localization, especially when enzyme activation is localized to an area such as focal adhesions, invadopodia, or lamellipodium. To better conjecture where this co-regulation is occurring we looked at additional robust associations with ENO1 phosphorylation and found in addition to paxillin and cortactin this site is most closely associated with phosphorylation on a protein called AFAP1L2, which stands for actin filament associated protein *like*-2. Despite several other phosphorylation sites on focal adhesion proteins, including integrin 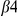 (ITGB4), p130Cas/BCAR1, and focal adhesion kinase (FAK), none of these sites is similarly regulated. Additional evidence supporting this is that the parental EGF subset of the *HER2* dataset also indicates that ENO1 and paxillin phosphorylation are tightly coupled with a third protein, Annexin A2, which has been implicated in cell spreading and migration [35] as well as formation of invadopodia [36]. Finally, a recent proteomic study found enolase is enriched in invadopodia [37] further supporting the hypothesis that co-regulation of enolase, paxillin, and cortactin phosphorylation is through shared cellular localization.

The temporal association of ENO1 phosphorylation with that of cortactin and paxillin is intriguing. ENO1, in full and dimeric form, is a metabolic enzyme. However, a short isoform of the same gene product has been shown to block Myc transcription factor activity by binding the Myc promoter [38]. This begs the question of whether ENO1 is playing a role in lamellipodium, invadopodia, or focal adhesions, related to its metabolic activity or some other gene product of yet to be discovered functionality. ENO1 phosphorylation on Y44 has long been known and when originally studied did not show a noticeable affect directly on its catalytic activity [34]. However, in these studies it was observed that only a small percentage, roughly 5%, of total enolase was phosphorylated and so catalytic differences due to phosphorylation may have been imperceptible. Perhaps compartmentalization of a fraction of altered enzymatic activity could play an important functional role; this activity may be confined to lamellipodia, invadapodia, or focal adhesions. Despite the excess of ATP in the cell, the induction of an ATP gradient within invadopodia could conceivably act as a method of invadopodia formation, since many components, such as F-actin, would be sensitive to a shift in the ATP to ADP ratio. There are other indications that phosphorylation of metabolic enzymes may indeed be playing a functional role and driving tumor progression [39] and our results may indicate a role for enolase phosphorylation specifically in the metastatic potential of tumor progression.

Finally, we found that using the concepts developed in MCAM, we could compare independent measures of the ERBB network and dissect signaling alterations occurring between different perturbations of the network, including ligand and receptor level differences. Using the co-occurrence matrix we were able to look “globally” at the differences between the networks. This study indicated that despite HER2 amplification, EGF stimulation drove signaling that was more similar between the two cell lines than it was for the same cell line under two different ligand stimulations. The greatest difference in signaling occurred when EGF stimulation of wild type cells was compared with HRG stimulation of HER2 overexpressing cells. We can dissect the signaling differences further by looking for those associations that have the most extreme differences between conditions. When we did this, we were fascinated to find that in the presence of HER2 overexpression, EGF drives very different dynamics on two phosphorylation sites of the adaptor protein SHC1 and sites on focal adhesion protein p130Cas/BCAR1. SHC1 is known to be recruited to EGFR by two different phosphopeptide binding events and it subsequently recruits, and activates, members of the MAPK cascade. Specifically, both Y349 and Y427 phosphorylation on SHC1 has been shown to recruit Grb2 [40]. Through its SH2 domain, SHC1 is recruited to EGFR Y1197 phosphorylation and through its PTB domain, it is recruited to EGFR Y1172 phosphorylation [41]. Therefore, it is no surprise that subsequent phosphorylation of SHC1 on Y427 and Y349 would be tightly co-regulated with that of EGFR Y1172 and Y1197 phosphorylation when the network is stimulated with EGF. However, what is surprising is this co-regulation is broken for only Y349 in the presence of HER2 overexpression. Instead of being most closely co-regulated with the receptor phosphorylations it is instead most closely co-regulated with Y228 phosphorylation on catenin delta-1, CTTND1, a protein known to be interact with E-cadherin at cell-cell junctions [42]. The authors of the original study found that HER2 amplification drives a higher migratory potential and posited that breakup of E-cadherin junctions would be essential to this process. Our finding may therefore indicate that SHC1 plays an important role in this process and that the differential regulation of Y349 and Y427 is perhaps driven by two populations of SHC1, one of which is localized at cell-cell junctions and which is differentially regulated, indicated by the sustained phosphorylation of SHC1 Y349 relative to Y427 phosphorylation, and a second population that is recruited to the receptor, which is probably the dominant population in EGF stimulation of wild type HMECs. The sequence surrounding catenin delta-1 Y228 matches known preferences for SHC1 SH2 recognition. In addition, multiple sites on the focal adhesion protein p130Cas/BCAR1 experience differential regulation in the presence of HER2 overexpession. Increased migration would come as a result of the disruption of both cell-cell contacts and cell-substrate contacts, so these sites might indicate a particular role in how cell-substrate contacts are disrupted in HER2 overexpressing cells. All of these data may help us in understanding the aggressiveness of tumor cells with HER2 amplification.

The methodology developed here has wide applicability to data mining of all varieties. Permutations of clustering parameters and their judgement of success by pertinent categorical data has the capability of producing a wide array of solutions that together span a meaningful range of data separation. MCAM, as developed, can be applied directly to any dataset currently in the PTMScout database, and any proteomic dataset that measures phosphorylation or lysine acetylation can be added by the public to PTMScout for analysis by MCAM. Extension to any type of multidimensional biological measurements simply requires the alteration of the target categorical data. For example, in addition to using gene and protein annotation information, one could look for known transcription factors when mining gene expression data. Another benefit of MCAM is that it provides a method for comparing the relationship between independent measurements of a system, even if the overlap of measurements is incomplete. This methodology provides a new method for understanding the relationship of quantitative measurements with each other, and importantly provides a means in which to judge the outcome of a parameter of clustering with regards to resulting power of inference. This is a much-needed tool when one lacks a satisfactory ‘gold standard’ by which to evaluate the impact of various parameters of unsupervised learning.

## Materials and Methods

### Dataset preparation and clustering

The *EGF4* and *HER2* datasets were loaded into PTMScout [24] and then subsequently reassigned to default assignments through the PTMScout ‘ambiguity’ interface to ensure all isoform selections represent the best overlap with current annotations. The default dataset was then reloaded into PTMScout. This modified dataset was then exported from PTMScout's ‘subset evaluation’ page as a tab-separated file, which was then loaded into Matlab for clustering.

The flat text file of the dataset was imported into Matlab based on DataRail object structures [43]. Transforms, distance metrics, and algorithms are from the Matlab environment and its toolboxes, downloaded from other resources, or developed for our purposes. Ncut code for Matlab was obtained from http://www.cis.upenn.edu/~jshi/software/ based on the algorithm description in [44], affinity propagation (AP) clustering code was downloaded from http://www.psi.toronto.edu/affinitypropagation/software/apcluster.m based on the algorithm described in [18]. A self organizing map (SOM) Matlab toolbox was downloaded from http://www.cis.hut.fi/somtoolbox/ and is based on the algorithm described in [45]. Affinity propagation clustering was modified to accept an arbitrary distance metric, but does not accept an argument for K. SOMs only utilize the Euclidean distance metric. Average linkage distance is used in hierarchical clustering. Kmeans uses the squared value of the Euclidean distance and does not accept the Chebychev distance metric. The largest value of K is bounded by a number that would produce roughly 5 phosphopeptides per cluster, assuming a solution were to equally distribute all phosphopeptides, which in this case is Kmax = 14. For non-deterministic algorithms, such as SOMs and Kmeans, we store the random seed so that results can be exactly reproduced, but allow the random seed to vary between individual implementations so as to ensure we do not force all implementations of the algorithm into a poorly performing local minima.

### Enrichment analysis and multiple hypothesis correction

Clustering assignments are written from Matlab into a tab-separated file, which is then loaded into PTMScout's ‘subset evaluation’ tool and enrichment is calculated on the PTMscout server using the ‘MCAM’ feature. Enrichment is calculated as in PTMScout's subset selection enrichment analysis, using a hypergeometric distribution calculation [24] to test for the overrepresentation of a label in a cluster compared to the full dataset. The MCAM feature has variable arguments, which were set as following: the motif algorithm branch cutoff is set at 1e-2 [46]; Scansite prediction levels of three and better are considered based on an empirical analysis of the tradeoff between the false positive rate and total hypothesis rejection (data not shown); Domain predictions of 1e-5 and more stringent are allowed; the Benjamini and Hochberg FDR procedure was used with an alpha value of 5e-2 [25]. False discovery rate correction (FDR) was performed at the metric and set levels as following: p-value calculations were accumulated for all tests within a category across a cluster set and the p-value satisfying an FDR alpha value of 0.05 was used to determine final enrichment for that metric. Three main data structures are translated from the PTMScout MCAM calculations into Matlab structures can then be used in Matlab for parameter refinement and analysis into results. Random controls were performed using two methods described in the results section. The first method randomized the data matrix and the second method randomized the metadata labels by reshuffling the vector to phosphopeptide mappings.

### Parameter refinement

Once enrichment structures are completed on the PTMScout server, they are loaded locally into Matlab. In order to determine parameters that produce clustersets with a relatively small degree of biological enrichment, we test the impact on the total enrichment when all clustersets generated with a particular parameter are removed from the full MCA. We perform this test systematically for every parameter used. Parameters whose removal improves the total enrichment across all categories by at least 2% without decreasing the power of enrichment in any single category by more than 10% are pruned to create 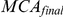. Where designated, in the HER2 dataset a total improvement of 3% was required to ensure the final sizes were roughly the same.

### Calculation of mutual information

In order to compare the clustering mappings of two clustering set solutions, we calculated the pairwise mutual information, I(X;Y), between clustering sets X and Y according to the equation given below, where X is composed of clusters 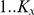 and Y is composed of clusters 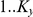, p(x,y) indicates the joint probability distribution function of X and Y, and 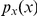 and 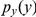 indicate the marginal probability distributions of X and Y, respectively.
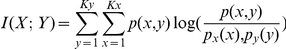



## Supporting Information

Figure S1 **The** ***EGF4*** **dataset projected onto the top three principal components.** The 77 phosphopeptide vectors were normalized to their maximum and then plotted on the first three principal components for illustration of the full multidimensional dataset.
(PDF)

Figure S2
**Enrichment and random controls for** 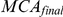 **using an alternate randomization process.** Procedures as described for Figure 2 were used to compare the real results of enrichment to random results, where randomization controls for the metadata labeling of phosphopeptide objects.(PDF)

Figure S3
**Comparison of Bonferroni and FDR correction methods on the enrichment and random controls for** 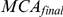 **.** Procedures as described for Figure 2 were used to compare the real results of enrichment to random results, for both FDR and Bonferroni correction methods with a target alpha of 0.05.(PDF)

Figure S4
**Enrichment and random controls for subsets of** 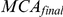 **.** A) The false positive rate and rates of enrichment and null hypothesis rejection when only cluster sets derived using the FFT transform are considered. An improvement in Scansite Kinase predictions is seen compared to the full 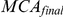. B) The zscore transform was chosen to create a comparable subset of solutions, and in contrast, here no improvement in Scansite Kinase terms is produced.(PDF)

Figure S5
**Histogram of biological label enrichment based on number of times they appear in** 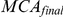 **.** The number of unique biological labels found enriched in 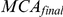 are given based on the number of times they occur across 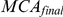. For example, there are 539 unique labels that occur at least once, no labels that occur in 100% of of the clustersets, and 39 labels that occur at least 25% of the time.(PDF)

Table S1
**Exploration of parameter relationships through mutual information.** Average MI is calculated for subsets of 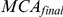 based on a single parameter type. Similarity between two parameters is also considered by finding the average MI of a subset containing only two parameter types. This supplementary information contains graphs and tables of the average MI by subset.(XLSX)

Table S2
**List of all enrichment produced in** 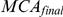 **and the number of times it appeared as enriched in the 331 sets.**
(XLSX)

Table S3
**Supplemental co-occurrence information.** List of all possible pairwise relationships between phosphopeptides in the *EGF4* and *HER2* analyses and the number of times they co-occur in their respective 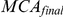. This also contains the listings for the three groups highlighted in Figure 4 C and D.(XLSX)

Table S4 **Supplemental ERBB dataset comparisons.** List of the sites that are in common between the EGF4 and HER2 datasets, correlations of the co-occurrence matrixes and lists of the extreme differences between sets highlighted by relationships moving from 75% or more co-occurrence to less than 25%.
(XLS)

## References

[1] Mann M, Jensen ON (2003). Proteomic analysis of post-translational modifications.. Nat Biotechnol.

[2] Oberprieler NG, Tasken K (2010). Analysing phosphorylation-based signalling networks by phospho ow cytometry.. Cell Signal.

[3] Emili AQ, Cagney G (2000). Large-scale functional analysis using peptide or protein arrays.. Nat Biotechnol.

[4] Choudhary C, Mann M (2010). Decoding signalling networks by mass spectrometry-based proteomics.. Nat Rev Mol Cell Biol.

[5] Yarden Y, Sliwkowski MX (2001). Untangling the ErbB signalling network.. Nat Rev Mol Cell Biol.

[6] Nolen B, Taylor S, Ghosh G (2004). Regulation of protein kinases: Controlling activity through activation segment conformation.. Mol Cell.

[7] Yaffe MB, Cantley LC (1999). Signal transduction. grabbing phosphoproteins.. Nature.

[8] Darnell J J E (1997). STATs and gene regulation.. Science.

[9] Diella F, Gould CM, Chica C, Via A, Gibson TJ (2008). Phospho.ELM: a database of phos-phorylation sites–update 2008.. Nucleic Acids Res.

[10] Olsen JV, Blagoev B, Gnad F, Macek B, Kumar C (2006). Global, in vivo, and sitespecific phosphorylation dynamics in signaling networks.. Cell.

[11] Zhang Y, Wolf-Yadlin A, Ross PL, Pappin DJ, Rush J (2005). Time-resolved mass spectrometry of tyrosine phosphorylation sites in the epidermal growth factor receptor signaling network reveals dynamic modules.. Mol Cell Proteomics.

[12] Wolf-Yadlin A, Hautaniemi S, Lauffenburger DA, White FM (2007). Multiple reaction monitoring for robust quantitative proteomic analysis of cellular signaling networks.. Proc Natl Acad Sci U S A.

[13] McCallum J, Ganesh S (2003). Text mining of DNA sequence homology searches.. Appl Bioinformatics.

[14] Tavazoie S, Hughes JD, Campbell MJ, Cho RJ, Church GM (1999). Systematic determination of genetic network architecture.. Nat Genet.

[15] Li X, Lu X, Tian J, Gao P, Kong H (2009). Application of fuzzy c-means clustering in data analysis of metabolomics.. Anal Chem.

[16] Eisen MB, Spellman PT, Brown PO, Botstein D (1998). Cluster analysis and display of genomewide expression patterns.. Proc Natl Acad Sci U S A.

[17] Tamayo P, Slonim D, Mesirov J, Zhu Q, Kitareewan S (1999). Interpreting patterns of gene expression with self-organizing maps: methods and application to hematopoietic differentiation.. Proc Natl Acad Sci U S A.

[18] Frey BJ, Dueck D (2007). Clustering by passing messages between data points.. Science.

[19] Jain A, Murty M, Flynn P (1999). Data Clustering: A review.. ACM Computing Surveys.

[20] Priness I, Maimon O, Ben-Gal I (2007). Evaluation of gene-expression clustering via mutual information distance measure.. BMC Bioinformatics.

[21] D'Haeseleer P, Liang S, Somogyi R (2000). Genetic network inference: from co-expression clustering to reverse engineering.. Bioinformatics.

[22] van den Berg RA, Hoefsloot HC, Westerhuis JA, Smilde AK, van der Werf MJ (2006). Centering, scaling, and transformations: improving the biological information content of metabolomics data.. BMC Genomics.

[23] Giancarlo R, Scaturro D, Utro F (2008). Computational cluster validation for microarray data analysis: experimental assessment of Clest, Consensus Clustering, Figure of Merit, Gap Statistics and Model Explorer.. BMC Bioinformatics.

[24] Naegle KM, Gymrek M, Joughin BA, Wagner JP, Welsch RE (2010). PTMScout, a web resource for analysis of high throughput post-translational proteomics studies.. Mol Cell Proteomics.

[25] Benjamini Y, Hochberg Y (1995). Controlling the false discovery rate - a practical and powerful approach to multiple testing.. J Roy Stat Soc B.

[26] Zhang Y, Wolf-Yadlin A, White FM (2007). Quantitative proteomic analysis of phosphotyrosine-mediated cellular signaling networks.. Methods Mol Biol.

[27] Tashiro K, Tsunematsu T, Okubo H, Ohta T, Sano E (2009). GAREM, a novel adaptor protein for growth factor receptor-bound protein 2, contributes to cellular transformation through the activation of extracellular signal-regulated kinase signaling.. J Biol Chem.

[28] Gensler M, Buschbeck M, Ullrich A (2004). Negative regulation of HER2 signaling by the PEST-type protein-tyrosine phosphatase BDP1.. J Biol Chem.

[29] Mitra SK, Hanson DA, Schlaepfer DD (2005). Focal adhesion kinase: in command and control of cell motility.. Nat Rev Mol Cell Biol.

[30] Oser M, Condeelis J (2009). The cofilin activity cycle in lamellipodia and invadopodia.. J Cell Biochem.

[31] Badowski C, Pawlak G, Grichine A, Chabadel A, Oddou C (2008). Paxillin phosphorylation controls invadopodia/podosomes spatiotemporal organization.. Mol Biol Cell.

[32] Bowden ET, Barth M, Thomas D, Glazer RI, Mueller SC (1999). An invasion-related complex of cortactin, paxillin and pkcmu associates with invadopodia at sites of extracellular matrix degradation.. Oncogene.

[33] Azuma K, Tanaka M, Uekita T, Inoue S, Yokota J (2005). Tyrosine phosphorylation of paxillin affects the metastatic potential of human osteosarcoma.. Oncogene.

[34] Cooper JA, Esch FS, Taylor SS, Hunter T (1984). Phosphorylation sites in enolase and lactate dehydrogenase utilized by tyrosine protein kinases in vivo and in vitro.. J Biol Chem.

[35] Babbin BA, Parkos CA, Mandell KJ, Winfree LM, Laur O (2007). Annexin 2 regulates intestinal epithelial cell spreading and wound closure through Rho-related signaling.. Am J Pathol.

[36] Hayes MJ, Moss SE (2009). Annexin 2 has a dual role as regulator and effector of v-Src in cell transformation.. J Biol Chem.

[37] Attanasio F, Caldieri G, Giacchetti G, van Horssen R, Wieringa B (2011). Novel invadopodia components revealed by differential proteomic analysis.. Eur J Cell Biol.

[38] Feo S, Arcuri D, Piddini E, Passantino R, Giallongo A (2000). ENO1 gene product binds to the c-myc promoter and acts as a transcriptional repressor: relationship with Myc promoter-binding protein 1 (MBP-1).. FEBS Lett.

[39] Hitosugi T, Kang S, Vander Heiden MG, Chung TW, Elf S (2009). Tyrosine phosphorylation inhibits PKM2 to promote the warburg effect and tumor growth.. Sci Signal.

[40] Ravichandran KS (2001). Signaling via Shc family adapter proteins.. Oncogene.

[41] Batzer AG, Rotin D, Urena JM, Skolnik EY, Schlessinger J (1994). Hierarchy of binding sites for Grb2 and Shc on the epidermal growth factor receptor.. Mol Cell Biol.

[42] Davis MA, Ireton RC, Reynolds AB (2003). A core function for p120-catenin in cadherin turnover.. J Cell Biol.

[43] Saez-Rodriguez J, Goldsipe A, Muhlich J, Alexopoulos LG, Millard B (2008). Flexible informatics for linking experimental data to mathematical models via DataRail.. Bioinformatics.

[44] Shi J, Mailik J (2000). Normalized cuts and image segmentation.. IEEE Trans Pattern Anal Mach Intell.

[45] Kohonen T (1990). The self-organizing map.. Proceedings of the IEEE.

[46] Joughin BA, Naegle KM, Huang PH, Yaffe MB, Lauffenburger DA (2009). An integrated comparative phosphoproteomic and bioinformatic approach reveals a novel class of MPM-2 motifs upregulated in EGFRvIII-expressing glioblastoma cells.. Mol Biosyst.

