# Supplementary figures and images for: MCAM: Multiple Clustering Analysis Methodology for Deriving Hypotheses and Insights from High-Throughput Proteomic Datasets
Source: PLoS Comput Biol. 2011 Jul 21;7(7):e1002119. doi: 10.1371/journal.pcbi.1002119 (PMC3140961; doi:10.1371/journal.pcbi.1002119)

EGF4 dataset projected onto first three principle components

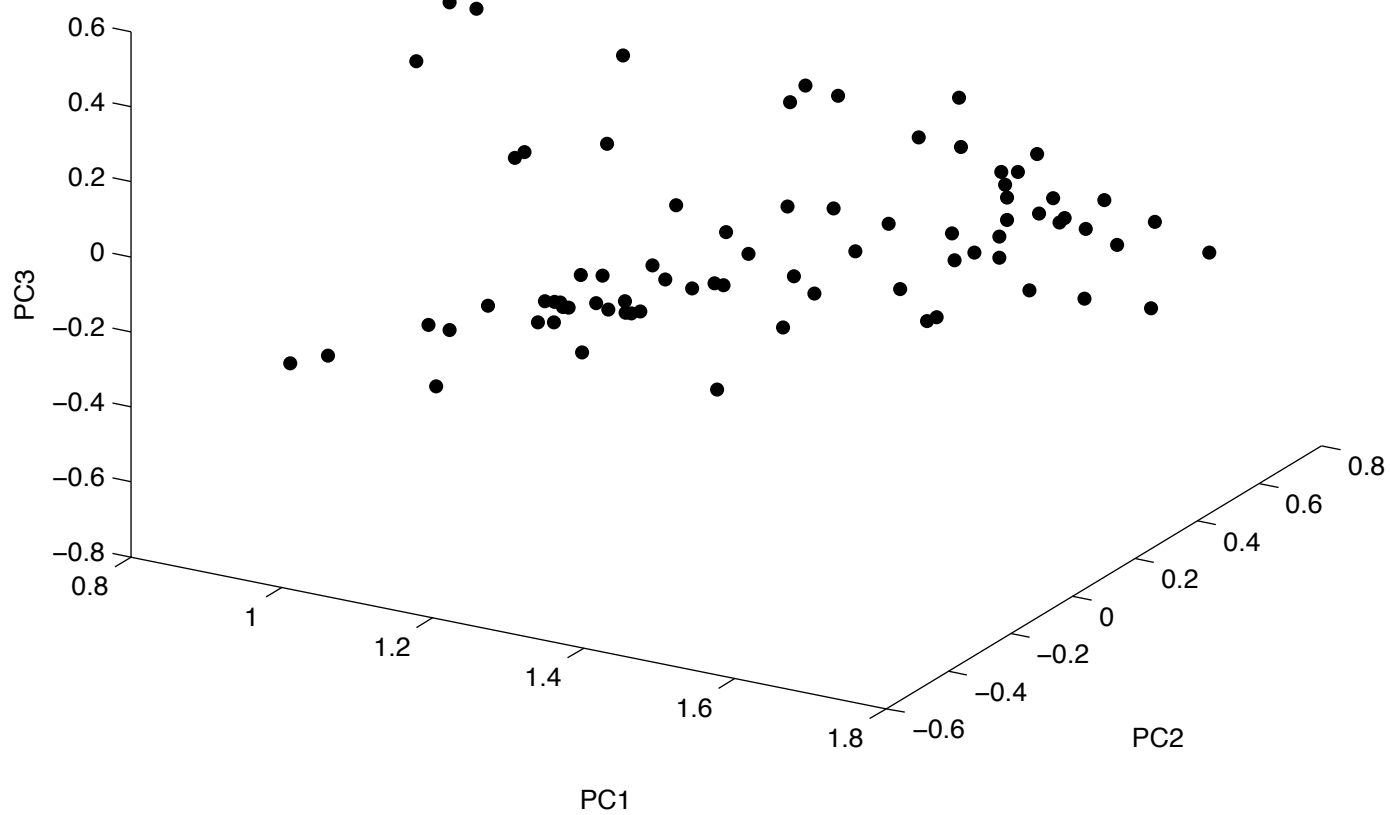

Supplement: Figure S1 — The EGF4 dataset projected onto the top three principal components. The 77 phosphopeptide vectors were normalized to their maximum and then plotted on the first three principal components for illustration of the full multidimensional dataset. (PDF) [file pcbi.1002119.s001.pdf]

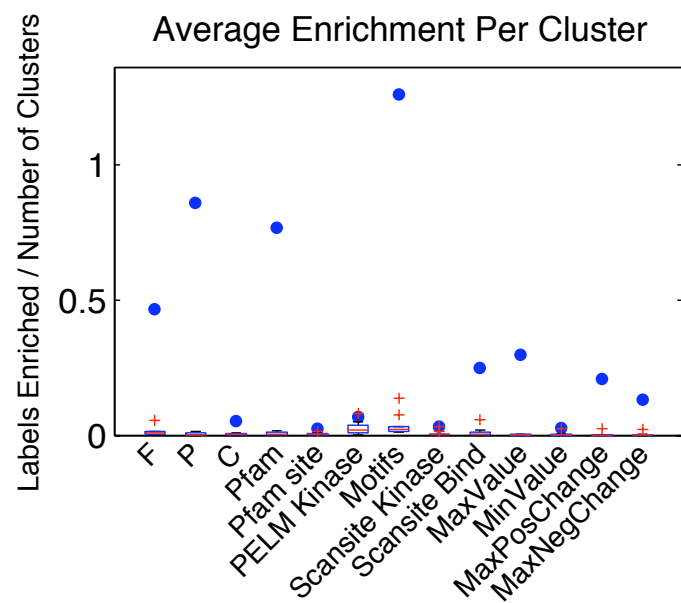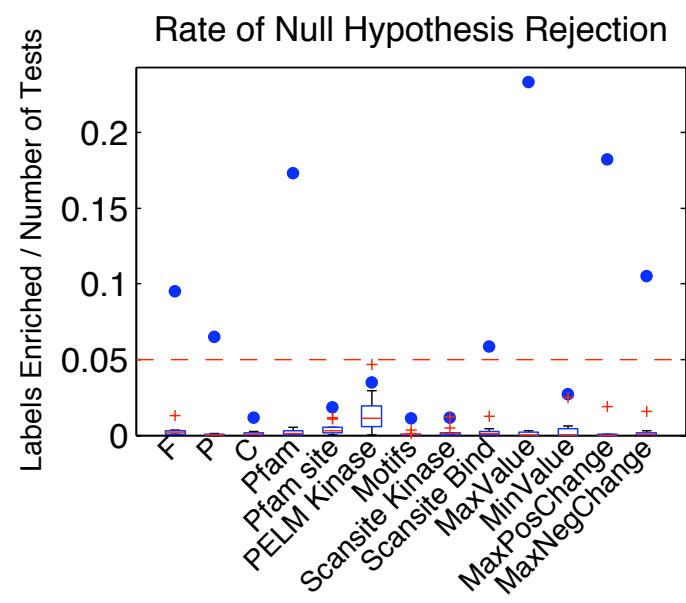

Supplement: Figure S2 — Enrichment and random controls for using an alternate randomization process. Procedures as described for Figure 2 were used to compare the real results of enrichment to random results, where randomization controls for the metadata labeling of phosphopeptide objects. (PDF) [file pcbi.1002119.s002.pdf]

## Bonferroni correction, $\alpha=0.05$

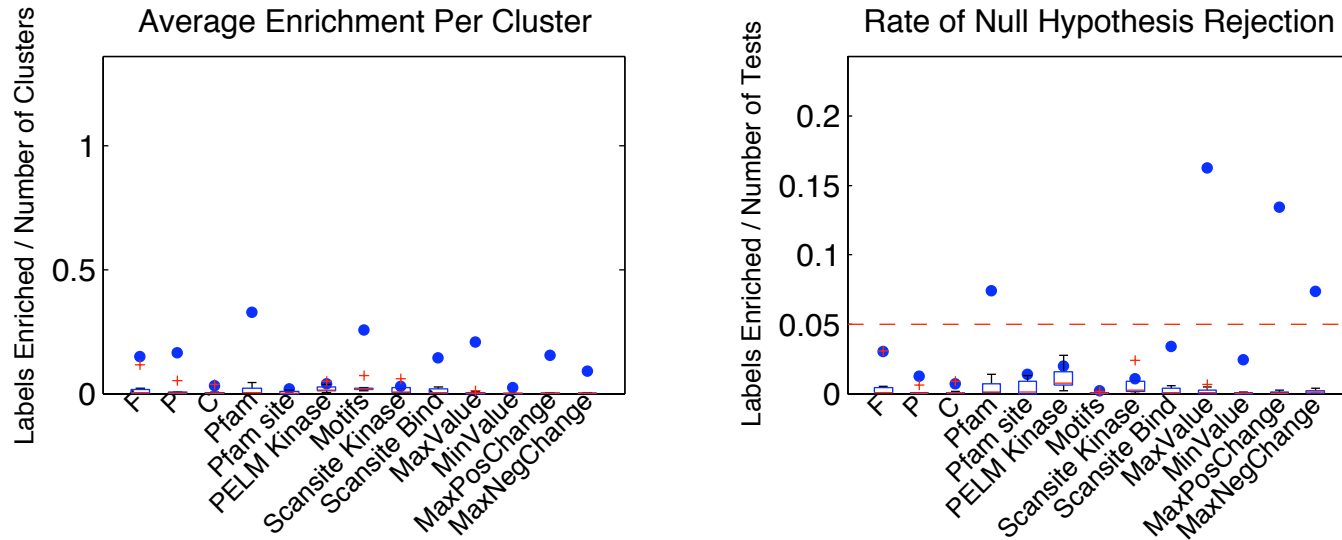

## False Discovery Rate correction, $\alpha = 0.05$

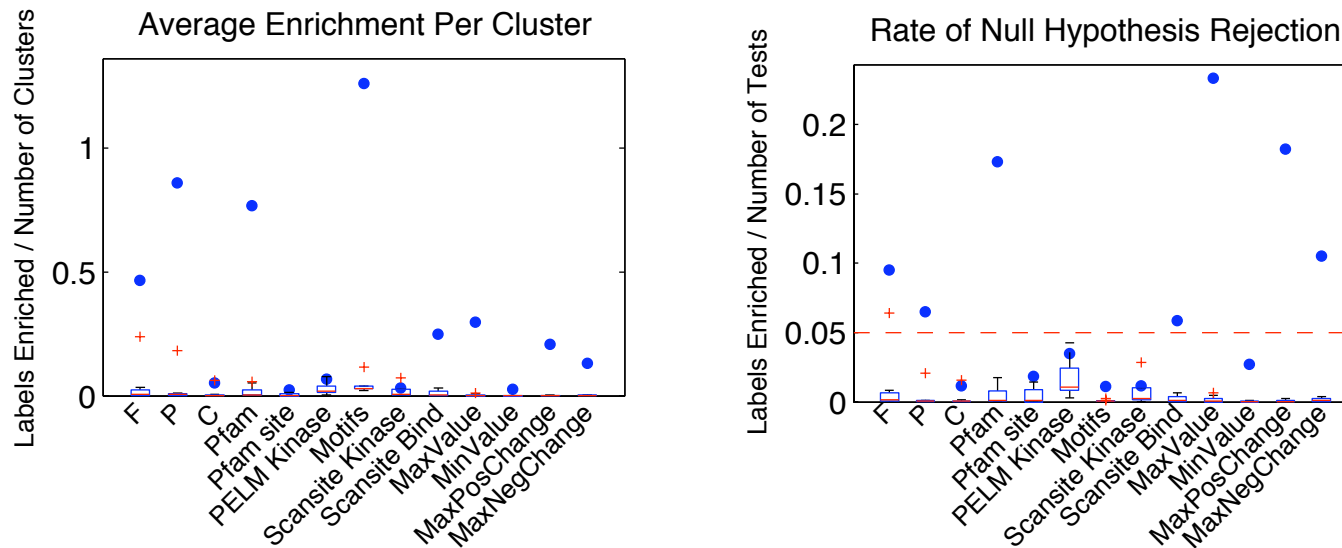

Supplement: Figure S3 — Comparison of Bonferroni and FDR correction methods on the enrichment and random controls for . Procedures as described for Figure 2 were used to compare the real results of enrichment to random results, for both FDR and Bonferroni correction methods with a target alpha of 0.05. (PDF) [file pcbi.1002119.s003.pdf]

A

FFT Clusters Only

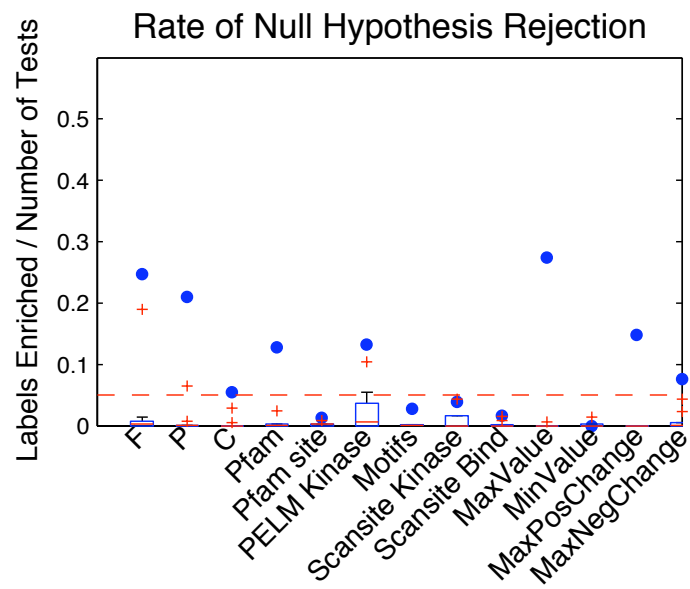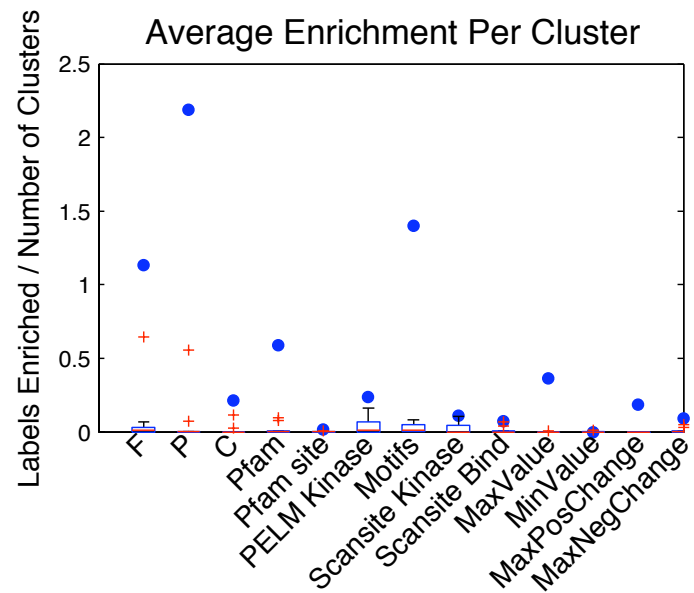

B

Zscore Clusters Only

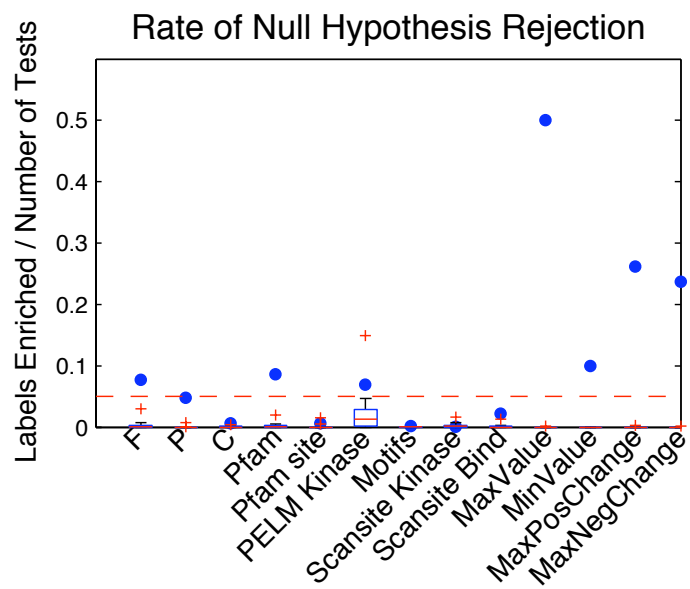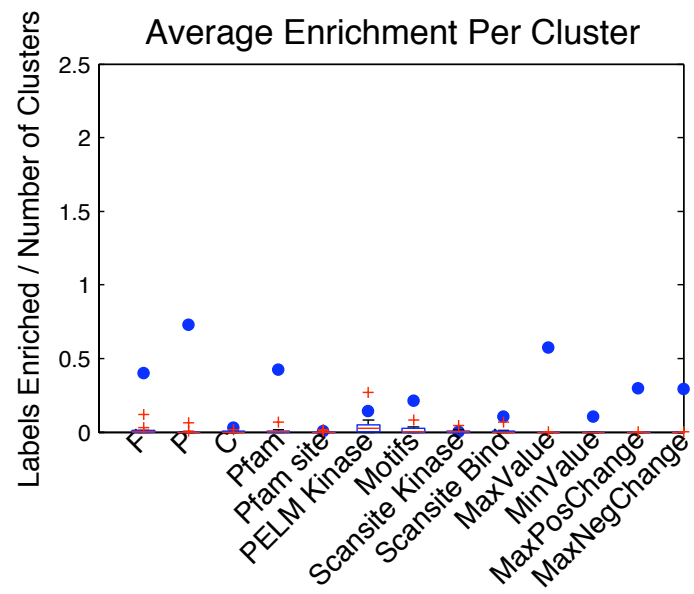

Supplement: Figure S4 — Enrichment and random controls for subsets of . A) The false positive rate and rates of enrichment and null hypothesis rejection when only cluster sets derived using the FFT transform are considered. An improvement in Scansite Kinase predictions is seen compared to the full . B) The zscore transform was chosen to create a comparable subset of solutions, and in contrast, here no improvement in Scansite Kinase terms is produced. (PDF) [file pcbi.1002119.s004.pdf]

Histogram of biological label enrichment

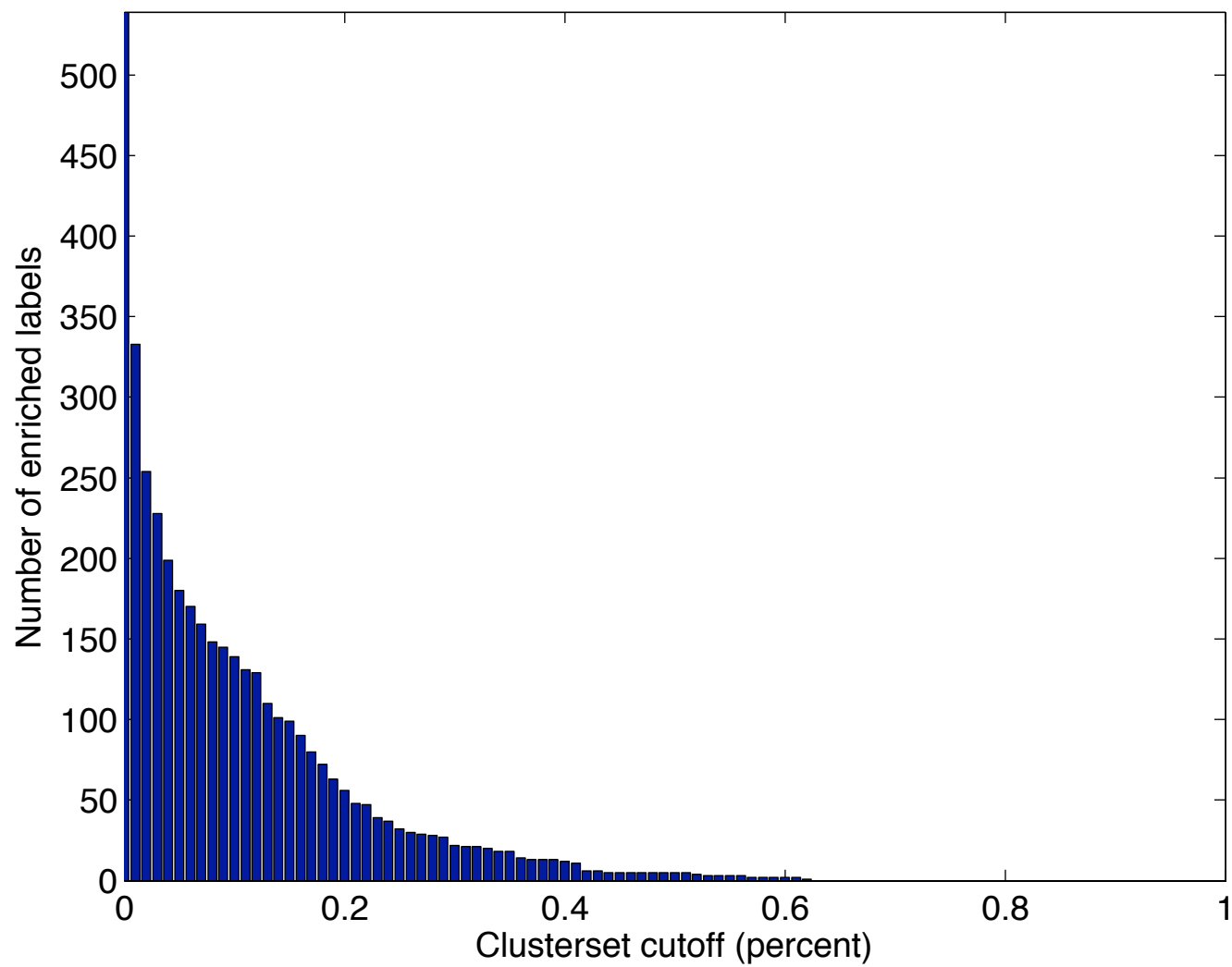

Supplement: Figure S5 — Histogram of biological label enrichment based on number of times they appear in . The number of unique biological labels found enriched in are given based on the number of times they occur across . For example, there are 539 unique labels that occur at least once, no labels that occur in 100% of of the clustersets, and 39 labels that occur at least 25% of the time. (PDF) [file pcbi.1002119.s005.pdf]
